# Particle coating alters mucociliary transit in excised rat trachea: A synchrotron X-ray imaging study

**DOI:** 10.1038/s41598-019-47465-1

**Published:** 2019-07-29

**Authors:** Mark Gardner, Alexandra McCarron, Kaye Morgan, David Parsons, Martin Donnelley

**Affiliations:** 1 Robinson Research Institute, University of Adelaide, North Adelaide, SA 5006 Australia; 2 Adelaide Medical School, University of Adelaide, Adelaide, SA 5000 Australia; 3 Respiratory and Sleep Medicine, Women’s and Children’s Hospital, 72 King William Road, North Adelaide, SA 5006 Australia; 4 School of Physics, Monash University, Clayton, Vic, 3800 Australia; 5 Institute for Advanced Study, Technische Universität München, Garching, Germany; 6 Chair of Biomedical Physics and Munich School of BioEngineering, Technische Universität München, Garching, 85748 Germany

**Keywords:** Imaging, Preclinical research

## Abstract

We have previously developed non-invasive *in vivo* mucociliary transport (MCT) monitoring methods using synchrotron phase contrast X-ray imaging (PCXI) to evaluate potential therapies for cystic fibrosis (CF). However, previous *in vivo* measurements of MCT velocity using this method were lower than those from alternate methods. We hypothesise this was due to the surface chemistry of the uncoated particles. We investigated the effect of particle surface coating on MCT marker performance by measuring the velocity of uncoated, positively-charged (aminated; NH_2_), and negatively-charged (carboxylated; COOH) particles. The effect of aerosolised hypertonic saline (HS) was also investigated, as previous *in vivo* measurements showed HS significantly increased MCT rate. PCXI experiments were performed using an *ex vivo* rat tracheal imaging setup. Prior to aerosol delivery there was little movement of the uncoated particles, whilst the NH_2_ and COOH particles moved with MCT rates similar to those previously reported. After application of HS the uncoated and COOH particle velocity increased and NH_2_ decreased. This experiment validated the use of COOH particles as MCT marker particles over the uncoated and NH_2_ coated particles. Our results suggest that future experiments measuring MCT using synchrotron PCXI should use COOH coated marker particles for more accurate MCT quantification.

## Introduction

The mucociliary transport (MCT) system is an important part of the body’s immune system, enabling pathogens, inflammatory cells and foreign particles that are inhaled into the lungs to become captured in the mucus, transported up the airways and trachea, and then swallowed and excreted or coughed clear^1^. MCT is facilitated by the action of cilia, microscopic hair-like structures that beat in a coordinated fashion to move mucus out of the lungs. By measuring the MCT rate, the health and performance of the MCT system can be evaluated^2^. This is a useful tool for diseases such as Cystic Fibrosis (CF), in which a mutation in the cystic fibrosis transmembrane conductance regulator (*CFTR*) gene and subsequent protein results in airway dehydration^3^ and inhibited mucus clearance. The inhibited clearance enables infection, inflammation, and destruction of lung tissue^4^. Direct assessment of localised improvements in MCT in CF animal models – for example, in response to a *CFTR* airway gene therapy^5^– might provide a more rapid and targeted therapy-assessment than evaluation of whole lung changes, such as standard pulmonary function testing (e.g. FEV_1_), that may be insensitive to beneficial small changes and might take months to years to appear.

Most MCT assessment methods involve depositing MCT marker particles onto the mucus on the airway surface, and then tracking their motion over time. Previous MCT assessments in animal models have used a range of methodologies. Examples of these include computed tomographic (CT) imaging to assess the motion of individual large 350 μm tantalum disks in pig airways^6^; gamma scintigraphy to quantify the bulk clearance of radiolabelled particles from the mouse nose^7^; and a dissecting microscope and filter to assess the trans-tracheal bulk motion of fluorescent particles^8^. However, each of these methods has limitations, including being invasive, having low resolution, or not being able to be performed *in vivo*. Our group has developed synchrotron propagation-based phase contrast X-ray imaging (PCXI) based methods for measuring the *in vivo* MCT rate in real-time in a range of animal models from mice^9,10^ to pigs^11^, with particles ranging in size from 20 μm in diameter up to 200 μm, respectively. With PCXI the individual particle motions can be non-invasively tracked over time, and the spatial and temporal variations in MCT behaviour across the airway surface can be characterised, including their responses to different pharmaceutical and genetic therapies.

The application of aerosolised hypertonic saline (HS) to the airway surface has been shown to increase the mucus clearance rate^9,12^, significantly reduce the number of pulmonary exacerbations, and improve traditional lung function parameters (i.e. FVC and FEV1)^13^. Inhaled HS changes the osmotic balance, drawing water from the epithelial layer onto the airway surface, leading to a temporary increase in the volume of the airway surface liquid (ASL) and improved mucus clearance. Given its ability to transiently restore mucus clearance, HS has proven useful in the clinical treatment of CF airway disease^12,13^. Previous studies investigating the effect of HS on lung function and mucus clearance rate have relied on a small number of MCT measurements with the time difference between measurements being of the order of several minutes^12^, or by using metrics from lung function tests^13–15^. Using the PCXI method for MCT monitoring described previously, the dynamic effect of HS on MCT rate has been investigated in live mice^9^ and pigs^11^, as well as in excised sheep trachea^16^.

Our previous PCXI studies used uncoated high refractive index (HRI) glass beads^10,11,16^ MCT marker particles. These beads are visible using PCXI, and have extremely uniform size, shape and surface properties, which we hypothesised would result in relatively uniform MCT behaviour. However, we found that 20–30 μm HRI marker particles are cleared at a lower rate than expected. We hypothesised that this was due to the surface properties of the beads, including surface charge, resulting in the beads “sticking” to the airway surface, resulting in ineffective clearance. There is research to suggest that nanoparticle surface properties can affect the performance of the MCT system^17,18^. Particle surface chemistry and charge are thought to also play an important role in mucin aggregation^19,20^ with MCT rate measurement studies typically performed using particles with a carboxyl-coating^21,22^ We hypothesise that altering our MCT assessment method to utilise carboxyl coated beads instead of uncoated beads will allow for a measured MCT marker velocity that is closer to MCT rates measured using alternate methods. Improved performance of the MCT marker particles will allow for further investigations into the dynamic effect of HS on airway surface rehydration and MCT rate in a range of CF animal models.

The aim of this study was to assess the variability in MCT rate resulting from the use of functionalised particles with different coatings, and to validate the use of carboxyl particles for future *in vivo* MCT measurement studies, including in CF rats^23^. The performance of negatively charged carboxyl particles was evaluated in comparison to the uncoated particles, and particles with a positively charged NH_2_ coating^19^. MCT measurements were made *ex vivo* using tracheas from normal rats. An *ex vivo* setup was used for this first investigative comparison, as *in vivo* measurements of MCT rate show much larger variability than *ex vivo* measurements, due to respiration and other motion artefacts.

## Results

Experiments were performed at the SPring-8 Synchrotron Radiation Facility in Japan. Tracheas were excised from rats and placed into a modified humidified tissue chamber that positioned the trachea in the path of the X-ray beam. We validated that the ventilated air has greater than 99% humidity, and that the air surrounding the trachea inside the chamber was at 99.02% humidity (SD 0.89, n = 3). MCT marker particles were either uncoated (n = 6), or carboxyl (n = 10), or NH_2_ (n = 5) coated, and were delivered to the surface of the trachea. Images were captured every 500 ms and the imaging period lasted for 20 minutes for each trachea. After 2 minutes of baseline imaging, HS aerosol was applied into the trachea.

### Mean particle speed

**Figure 1:**
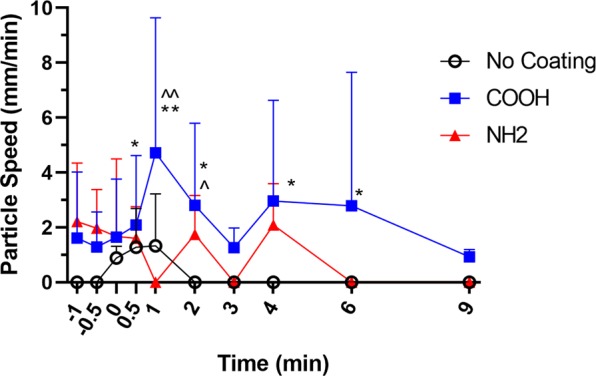
Average MCT rate of the different particles overtime (Mean ± SEM). Stationary particles were not included in this analysis. Timepoint 0 minutes indicates when the hypertonic saline was applied. Particle coating groups were no coating control (n = 6), COOH coating (n = 10) or NH_2_ coating (n = 5). Statistical analysis was conducted using a two-way reapeated measurements ANOVA with significance 0.05, with Tukey corrections for multiple comparisons. F values for time, treatment, and time vs treatment interaction were 0.9512, 2.636 and 0.4133 respectively *COOH > No Coating (p < 0.05),**COOH > No Coating (p < 0.0001), ^COOH > NH_2_ (p < 0.05), ^^COOH > NH_2_ (p < 0.001).

The performance of the different particle coatings was compared by manually tracking the particle velocity between consecutive frames for the different particle groups as described previously^9^. An example image showing the particles moving is shown in Supplementary Video S1. The average particle velocity of the non-stationary particles for each of the coating groups for the different time points is shown in Fig. 1. This figure shows that for the uncoated particles, particle movement occurred after the application of the HS aerosol. However, the effect was brief, and 2 minutes after the HS was applied no uncoated beads could be detected moving, suggesting that the uncoated beads only move in the presence of HS aerosol, and possibly indicating that they require the presence of bulk fluid to move on the airway surface.

Figure 1 also shows that at time points 0.5 and 1 minute after the HS aerosol was applied the average velocity of the COOH particles was significantly higher than the uncoated particles. The average velocity of the COOH particles at time points 2, 4 and 6 minutes after the HS was applied was also significantly larger than the average velocity of the uncoated particles, as for these time points there was no particle movement. There was also no movement of the uncoated particles prior to the application of the HS aerosol. The difference in the uncoated particle velocity and the average velocity of the COOH particles at the 9 minute time point was not statistically significant (even though the average COOH velocity was larger than zero), possibly due to the large variability in the particle velocity measurements and the lower statistical power of the measurements when the particles were not moving.

Similarly to the COOH particles, Fig. 1 shows that the NH_2_ particles moved prior to the application of the HS Aerosol. Indeed, there was no significant difference in the average particle velocity between the COOH particles and the NH_2_ particles for this time. However, there was a significant difference in the COOH and NH_2_ particle velocity at time points 1 and 2 minutes after the HS aerosol was applied. At the 1-minute time point, a significant difference in the average particle velocity was recorded as there was no detectable particle movement for the NH_2_ particles, and there was an increase in the velocity of the COOH particles. This result suggests that the application of HS to the airway surface could reduce the velocity of the NH_2_ particles, which is opposite to the effect on the COOH particles.

For both the COOH coated particles and the NH_2_ particles there was a decrease in the mean particle velocity at the 3-minute time point after the HS was applied, and then an increase in particle velocity in the following time points. This could be caused by particles that were dispersed lower in the airway than the particles initially in the frame (i.e. not present in the initial images) moving up into the field of view. Since the uncoated particles did not move without the application of HS, this phenomenon was not observed for the uncoated particles.

### Particle speed distribution

**Figure 2:**
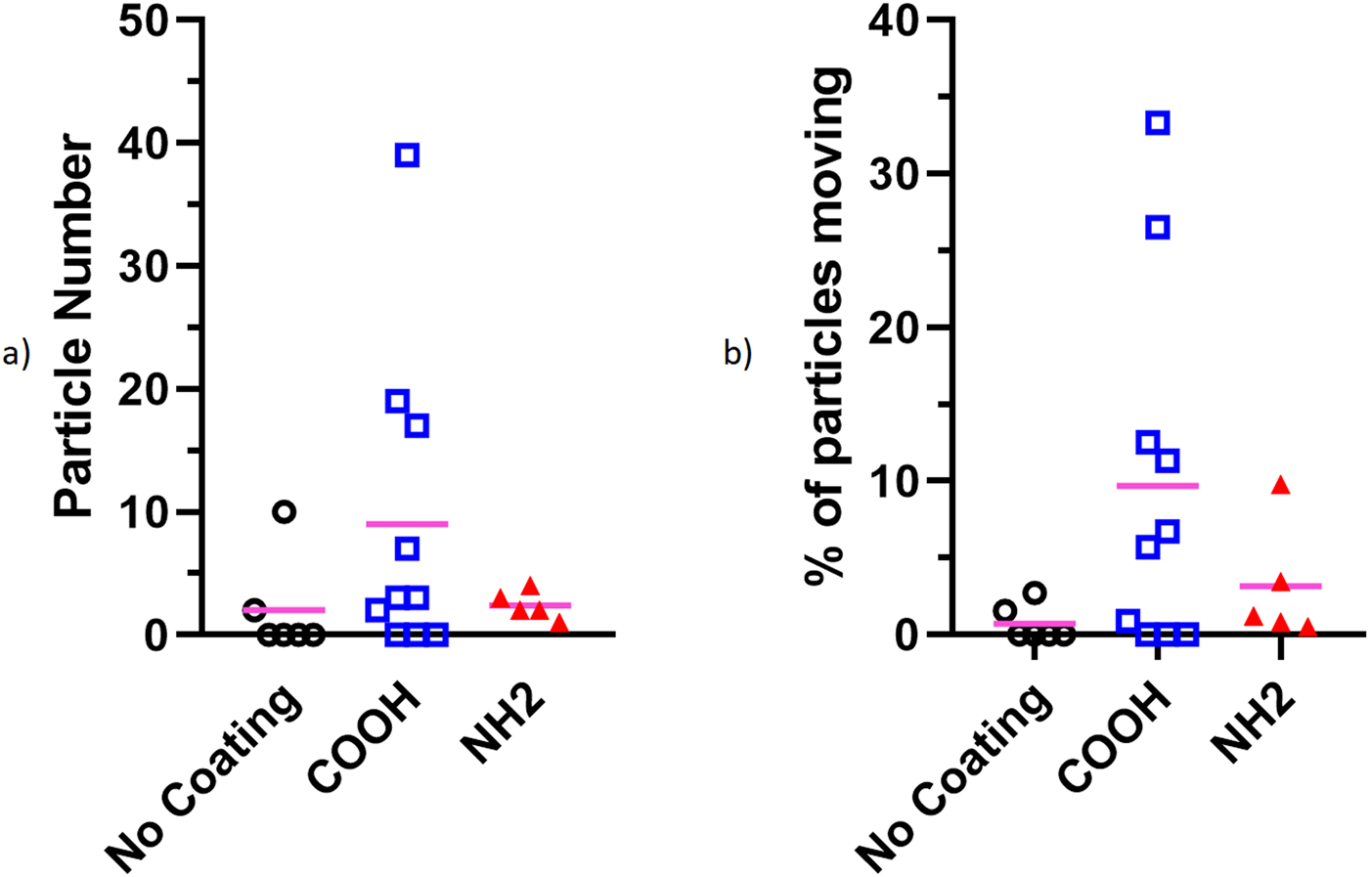
Maximum number of particles that were detected as moving (**a**) and expressed as a percentage of total particles detected in each frame (**b**). No significance reported between different groups. Statistical analysis was conducted using multiple t-tests with adjusted p-values using the Holm-Sidak method with significance p = 0.05.

The maximum number and percentage of particles that were classified as moving during analysis are shown in Fig. 2. This analysis was limited to the first 5 minutes of the image sequence, to limit it to particles that began in-frame. The average number and proportion of particles that moved was larger for the COOH particles, however this difference was not significant, which was determined using multiple t-tests with adjusted p-values using the Holm-Sidak method (significance p = 0.05). This lack of significance was most likely due to the large variability in the values and the relatively small number of particles in the field of view. These results also show that for most of the trachea samples only a small number of particles moved, and for some of the tracheas there was no significant observable particle motion (n = 4 for no coating, n = 3 for COOH coating). Additionally, for the uncoated particles, Fig. 2 shows that when particle movement did occur, the number and proportion of particles that did move was low.

**Figure 3:**
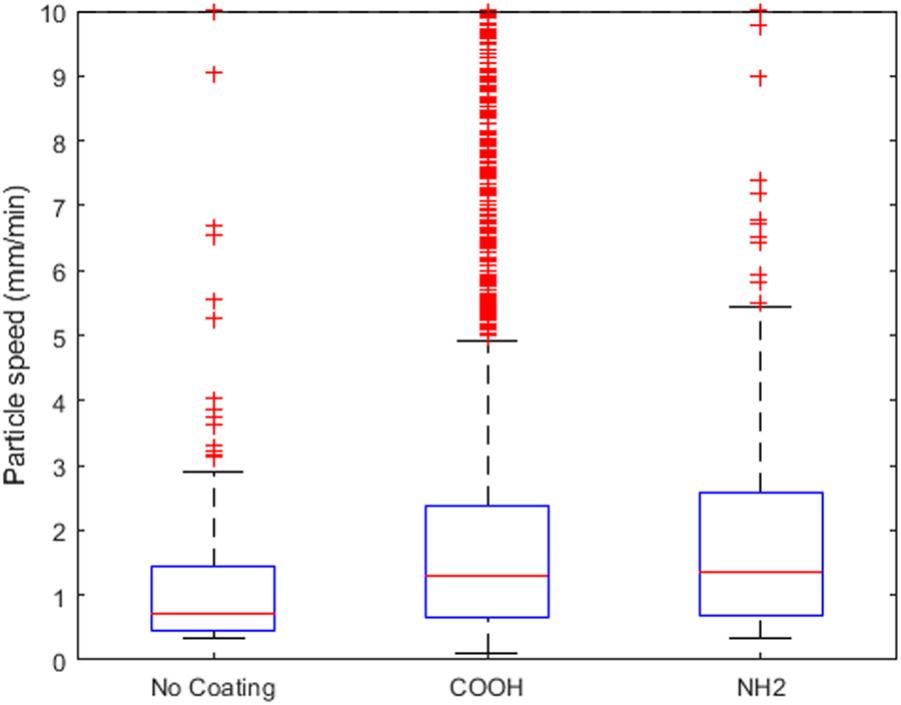
Distribution of the different measured particle velocity for the different particle coatings. Statistical outliers are indicated by the red crosses and are defined as q3 + 1.5 × IQR where q3 is the 75th percentile and IQR is the interquartile range.

Figure 3 shows the distribution of the different measured particle velocities from the different particle coatings. This figure demonstrates the large variability in the measured values, even within the same particle coating group. This result is consistent with our previous studies^9,16^, and is likely due to the complex and non-uniform nature of the MCT process, as well as variability between different animals. Similarly, Fig. 3 also shows the large number of outlier particle measurements (indicated as red crosses) that occurred, defined as q3 + 1.5 × IQR where q3 is the 75th percentile and IQR is the interquartile range. These statistical outliers also show the large variability in the measured particle velocities, caused by complex nature of the MCT process, as well as the variability between animals.

### Velocity heatmaps

**Figure 4:**
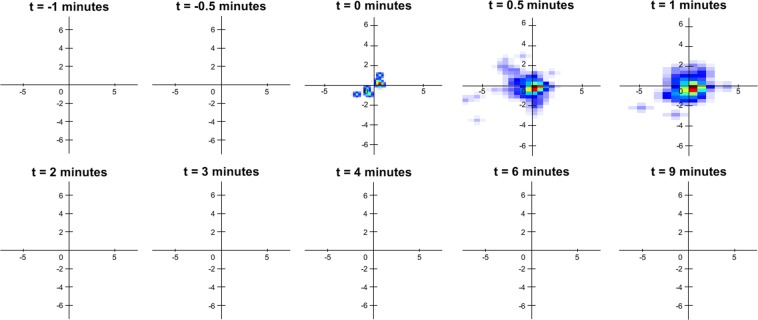
Velocity heat-map of the particles with no coating for the different time points where the particle velocity was analysed. The heat-map orientation matches the physical orientation of the trachea in the tissue bath, with the larynx to the left and lung to the right. Stationary particles are at the axis origin, slowly moving particles are close to the origin, and faster moving particles are further away. The angular position represents the particle direction of motion. Red and brown indicates more particles than yellow or blue.

**Figure 5:**
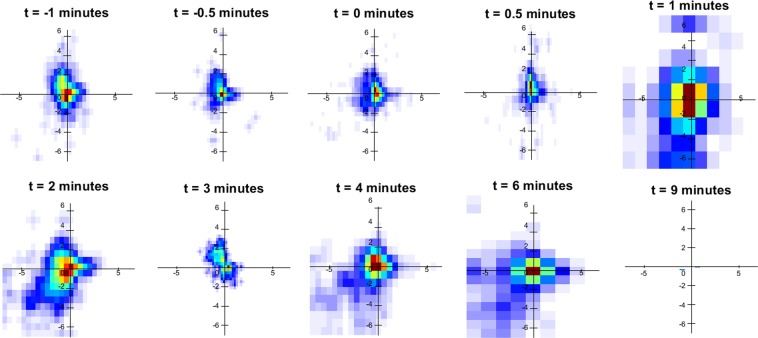
Velocity heat-map of the COOH coated particles for the different time points where the particle velocity was analysed.

**Figure 6:**
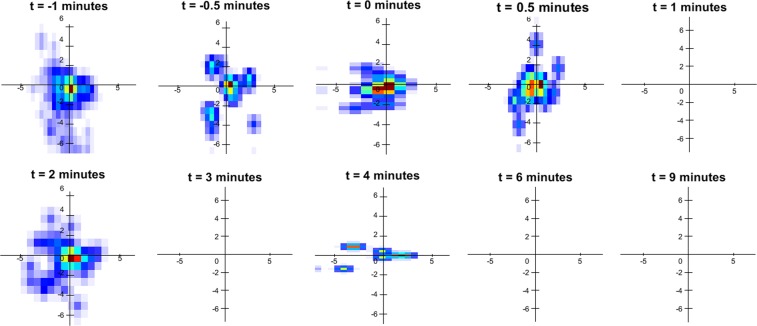
Velocity heat-map of the NH_2_ coated particles for the different time points where the particle velocity was analysed.

As well as comparing the absolute velocity magnitude of the different coated particles, velocity heat-maps were generated to help visualise the velocity profiles of the particles over different time points^16^. The ability to generate velocity heat-maps by analysing the behaviour of individual particles is a unique advantage of the synchrotron PCXI-based MCT assessment method. The velocity heat-map for the uncoated, COOH and NH_2_ particles are shown in Figs 4–6, respectively. In Fig. 4, the only time points at which a velocity profile could be generated for the uncoated particles were 0, 0.5 and 1 minute after the HS was applied. This was because in all other time points for all of the different trachea samples, there were no particles that were detected to be moving. Similarly, 2 minutes after the HS was added, there was also no movement detected in the uncoated particles, which was possibly when the bulk fluid effects of the HS disappeared. Similar to Fig. 1, these results suggest that the uncoated MCT marker particles will stick to the airway surface and remain stationary only to move if HS is added to the ASL. This result is in contrast to Figs 5 and 6, which show the velocity heat-maps of the COOH and NH_2_ particles respectively. In these figures MCT marker particle movement can be detected at the two time points before the HS is applied. Similarly, 2 minutes after the HS was added, there was still particle movement of the COOH particles, and some movement of the NH_2_ particles, suggesting that the COOH and NH_2_ particles can be used as MCT marker particles without adding HS to the airway surface.

The velocity heat-maps in Figs 5 and 6 show similar information to Fig. 1 regarding the differences between the mean particle velocity magnitudes of the NH_2_ and carboxyl coated MCT marker particles. However, the heat-maps make it clear that there is substantial heterogeneity in the particle movement direction as well as the velocity magnitude, which results in the low statistical power and large variability shown in Fig. 1. Prior to the application of the HS, there appears to be no significant difference between the velocity heat-maps in Figs 5 and 6. However, after the application of the HS, Fig. 6 shows a reduction in NH_2_ coated particle movement with minimal particle movement occurring, except for at the 2-minute time point. This is in contrast to the particle velocity heat-map shown in Fig. 5, which shows increased particle velocity and movement after the application of the HS (timepoints 1 and 2 minutes, and then sustained particle movement for the remainder of the experiment. Hence the addition of HS had opposite effects on the particle velocity of the carboxyl and NH_2_ coated particles, increasing the velocity of the carboxyl particles and decreasing the velocity of the NH_2_ particles.

## Discussion

A range of methods for monitoring MCT have been developed, but most are based on tracking the transit of some type of marker particle. The primary difference between these methods is the modality used to visualise particle motion. Hoegger *et al*. analysed the MCT rate of pigs using microdisks that were deposited via a catheter behind the vocal cords^6^. The position over time (and hence velocity) of the microdisks was measured using CT imaging, which both defined the very large disk size (to enable CT detection) and limited the time between successive images (~15 seconds). As a result, this method was well suited for assessing average MCT rates (of particles unlikely to be inhaled in normal life), but could not show faster changes in MCT rate effectively, such as the immediate change in MCT rate due to the introduction of a pharmacological substances^9^. Additionally, to be able to detect the particles using a standard human clinical CT system, the size of the microdisks were 350 × 25 μm, which is significantly larger than the thickness of the ASL^24^, and hence the microdisks would not be submerged in the ASL. This means that the velocity of the microdisks measured by Hoegger *et al*. may be less likely to represent the actual MCT rate than if the MCT marker particles that had a diameter closer to the ASL thickness.

Radioactive particles have also been used for MCT measurement using gamma scintigraphy. In this method radiolabelled particles are deposited into the airways, and their locations are measured using a gamma camera, which provides the position of particles when they undergo radioactive decay^7^. Rather than measuring the velocity and MCT behaviour of individual particles, this method measures the bulk amount of radiation present in different regions over time, and the time it takes to clear a certain percentage of the particles. In such bulk measures of particle motion the spatial and temporal resolution are low, but the advantages of this system are that it is relatively simple and readily translatable, having been implemented in mouse nasal airways through to humans^25^.

Fluorescent beads (100–200 nm) can also be used as MCT marker particles, making MCT measurement inexpensive and accessible^8^. However, the resolution of the images generated from the fluorescent beads does not allow for the measurement of individual MCT marker particle velocity, with MCT quantification only possible once the particles aggregate into “rafts” that are sufficiently large to be visualised. Additionally, Rogers *et al*. reported that to visualise the fluorescent beads, an incision had to be made to expose the trachea, so that the muscle and surrounding tissue did not impede the view. This makes their method more invasive than *in vivo* the PCXI methods described previously^9,11^, and can make longitudinal repeated-measures study designs (e.g. such as those described by Donnelley *et al*.^26^) impossible.

MCT rates can also be measured directly without the use of MCT marker particles through μOCT, a method that allows for high-resolution imaging of tissues such as airway epithelia^27^. This method also allows for direct measurement of the ASL depth. μOCT is often performed in excised tracheas, and as such, does not allow for *in vivo* MCT assessment, however newer μOCT methodologies are currently under development to overcome this limitation^28^.

Previous synchrotron PCXI-based MCT analysis experiments have used uncoated HRI marker particles in *in vivo* mouse^9^ and pig^11^ experiments, as well as in *ex vivo* sheep and pig experiments^16^. Following application of 22 μm uncoated HRI glass particles to normal and CF mouse nasal airways few particles moved^10^, and the measured particle velocity was lower than reported MCT rates in mice in other experiments^29^. *Ex vivo* MCT measurements in pig and sheep samples have also been reported using 103 μm uncoated HRI glass particles, with and without the effect of HS^16^. Those results showed some particle movement for the untreated baseline measurements (i.e. no HS added). A similar result was observed *in vivo* in pigs, which showed particle movement before the application of HS^11^. The beads used by Donnelley *et al*. for the pig and sheep MCT measurements were an order of magnitude larger in diameter than the beads used in the experiment described in this paper. The larger bead size may have made it more difficult for the particle to become ‘stuck’ to the airway surface, allowing for increased movement of the uncoated particles prior to the application of HS aerosol.

In the present *ex vivo* study the MCT rate was measured in excised rat tracheas by tracking the motion of deposited 20 μm particles. The effect of using different particle coatings was evaluated by comparing both the average and maximum particle velocities, as well as the number of particles that moved. The results demonstrated that the addition of COOH or NH_2_ coatings increased the average particle velocity (~2 mm/min) when compared with particles that had no coating (0 mm/min). Additionally, the results suggested that the performance of the COOH particles was different to the performance of the NH_2_ particles; application of HS to the airway surface reduced the velocity of the NH_2_ particles, which is opposite to the effect on the COOH particles. However, Chen *et al*. reported that positively charged nano-particles (i.e. with properties similar to the NH_2_ particle coating used here) could lead to mucin aggregation, and so the reduction in mean particle velocity after the application of the HS could be due to this effect^20^.

Henning *et al*. conducted an experiment looking at the effect of particle charge and other surface properties^17^. They suggested that the particle surface itself is more likely to affect the MCT rate than the particle surface charge, which is seemingly contradictory to the results in this paper. However, it is possible that the process of coating the alumina particles to apply a positive (or negative) charge altered the particle surface and this is responsible for the increase in MCT marker particle performance. This hypothesis would explain why the performance of both the COOH and NH_2_ particles are similar in the baseline imaging period.

The *ex vivo* results of this paper match previously measured rat *in vivo* MCT measurements by Tuggle *et al*.^30^ and Birket *et al*.^31^ using μOCT. The mean MCT rates for both the wild-type and CFTR^−/−^ rats that were 21 to 44 days postnatal were approximately 0.5 mm/min^30^. By 3 and 6 months of age, the mean MCT rate of the CFTR^−/−^ rats was slightly less than the 1 mm/min measured in the wild type rats^31^. These MCT rates measured by μOCT are similar to the baseline rates for the COOH and NH_2_ coated particles (Fig. 1, prior to the application of the HS solution) in the tracheas excised from 12 week old rats.

The results from the present study show a large amount of variability in the particle motion, likely because particles in varying locations within the airway move differently due to the presence of local mucus and different ciliation patterns. This observation is consistent with previous experiments measuring MCT rates using PCXI-based MCT assessment methods^9,11,16^, as well as other methods for monitoring MCT^30,32^. Although not statistically significant, the results in Fig. 2 suggest a trend towards more movement of COOH and NH_2_ coated particles than the uncoated particles, which would also increase the variability of the MCT measurements. This finding should be considered when designing future MCT rate experiments, particularly in regards to animal numbers to ensure significance in results.

We identified three limitations of this study. The primary limitation of this study was the small sample size, which was required due to the logistics of organising beamtime at a synchrotron facility. Given the large amount of variability in the particle velocity measurements (and hence the MCT rate) additional tracheal MCT assessments may have allowed for an improved comparison between the different particle coatings. Nonetheless, the study power was sufficient to demonstrate a difference between the coated and uncoated particles, particularly before the application of the HS aerosol.

The second limitation was that only female rats were used, which means differences in the stage of estrous cycle between animals could have influenced the MCT rates. It has been shown that sex hormones can influence the cilia beat frequency^33^, suggesting the varying estrogen and progesterone levels could be a contributing factor to the heterogeneity of the measured MCT marker particle velocities.

The third limitation was that after excision the tracheas were placed into PBS during preparation, which may have altered the airway surface liquid milieu. In future studies we plan to wrap the trachea in tissue soaked in buffer after excision, and place it in an oxygenated tube stored on ice.

Previous experiments using synchrotron PCXI to assess MCT behaviour were *in vivo* rather than *ex vivo*, but all utilised uncoated particles^9,11,16^. Hence the *ex vivo* results from this experiment suggest that the *in vivo* methods previously developed should be modified so that future experiments use COOH coated particles to maximise the accuracy and effectiveness of the experiments, which will allow for non-invasive and continuous monitoring of multiple individual MCT marker particle to measure the MCT rate of animals *in vivo*. The results of this paper also suggest that the NH_2_ particles could also be used as MCT marker particles in any future experiments that do not involve applying HS to modify the MCT rate. The manner in which the particle coatings interact with other therapies remains unknown.

Previous *in vivo* experiments using synchrotron PCXI to assess MCT behaviour were performed on mice^9^ or pigs^11^. However, the tracheas that were analysed in this paper were from rats, for which there are no published *in vivo* methods for MCT measurement. Future experiments will look at developing methods for monitoring the MCT rate in normal and CF rats *in vivo*, which will be adapted from our published methods, with the aim of being as equally as non-invasive and informative.

In this study, the particles were tracked using a manual tracking method. While this method has been successfully used in previous studies^9^, it is time consuming and subject to user analysis bias. To minimise such bias, manual tracking here (by MG) was blinded to the particle type and to the time point that was being observed. Future method development is planned to examine image analysis and automatic particle tracking methods that remove observer bias from these particle movement measurements.

## Conclusion

In summary, our findings have furthered the development of a previously reported method for MCT rate measurement by identifying improvements to particle MCT tracking ability. The addition of the NH_2_ or the COOH coatings to the particle surface increased particle velocity during the brief assessment period. The measured MCT rate increased for COOH coated particles with the addition of HS aerosol to the airway, when compared to the baseline MCT rate for the COOH particles. When compared with other studies, our findings here suggest that the measured velocity of the NH_2_ and COOH particles (as measured in the baseline imaging period) more closely resembles previously reported MCT rates in rats. Conversely, there was no significant movement of the uncoated particles until the HS aerosol was added to the airway, and when the uncoated particles did move, the measured particle velocity was significantly lower than the velocity of the COOH particles. Hence the use of COOH coated particles could lead to more accurate MCT rate measurements in future PCXI studies.

## Method

### Ethics statement

All animal studies were performed in accordance with protocols approved by the University of Adelaide (M-2017-113) and SPring-8 Synchrotron animal ethics committees.

### Imaging

Imaging was conducted at the BL20XU beamline at the SPring-8 Synchrotron radiation facility in Japan. The experimental hutch was located 245 m from the Synchrotron storage ring. The sample to detector distance was 11 cm, with a monochromatic beam energy of 25 keV.

Images were captured using a high-resolution X-ray converter (SPring-8 BM3) coupled to a sCMOS detector. The converter used a 10 μm thick scintillator (Gd_3_Al_2_Ga_3_O_12_) to convert X-rays to visible light, which was then directed to the sCMOS sensor using a x10 microscope objective lens (NA 0.3). The sCMOS detector was an Orca-Flash4.0 (Hammamatsu Photonics, Japan) with an array size of 2048 × 2048 pixels and a 6.5 × 6.5 µm native pixel size. This setup resulted in an effective isotropic pixel size of 0.53 µm and a field of view of 1.1 mm × 1.1 mm. An exposure length of 25 ms was chosen to maximise the signal to noise ratio of the beads in the airway^9^. A fast X-ray shutter was placed into the X-ray path to limit radiation dose by blocking the X-ray beam between exposures^9^.

### Sample preparation

Wild-type female albino Wistar rats (~12 weeks of age, ~200 g, n = 21) were used for all experiments. Rats were anaesthetised with 0.4 mL/100 g body weight of medetomidine (Domitor®, 0.06 mg/mL, Zenoaq, Japan), midazolam (0.8 mg/mL, Dormicum®, Astellas Pharma, Japan), and butorphanol (Vetorphale®, 1 mg/mL, Meiji Seika Pharma, Japan) via intraperitoneal injection. Once anaesthetised, the rats were humanely killed using intracardiac injection of 0.1 mL sodium pentobarbital (Somnopentyl, 64.8 mg/mL, Kyoritsu Seiyaku Corporation, Japan). The trachea was then excised from the carina to the cricoid cartilage, and the larynx was removed. The trachea was stored in warm phosphate buffered saline (PBS) during preparation.

**Figure 7:**
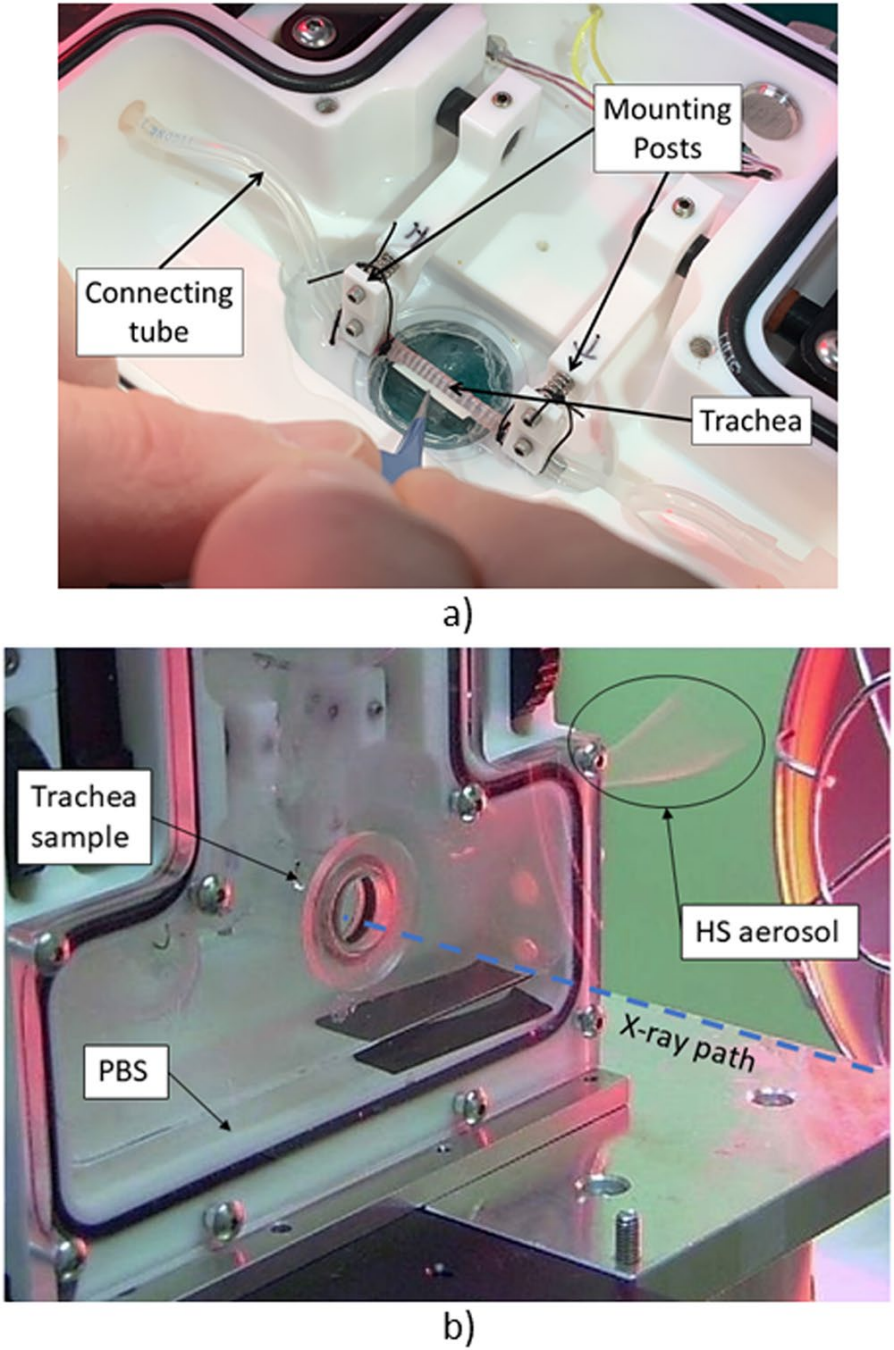
(**a**) Excised trachea placement in the Living Systems water bath. (**b**) Webcam image of the tissue bath on the sample stage in the imaging hutch. Note the PBS in the bottom of the bath, and the HS aerosol coming out of the right side of the bath during imaging.

The excised trachea was set up in a sealed tissue chamber (Living Systems CH-1, USA) that had been modified to add a reduced depth x-ray imaging section to minimise the amount of x-ray absorption during the short, high x-ray flux exposures (see Fig. 7). A suture was applied around the proximal end of the trachea (on the left side in Fig. 7a) to secure and seal it to the connecting tube. Cartilage rings were then removed from the distal end of the trachea, until the trachea sample was able to fit between the two mounting posts without being stretched. The trachea was then aligned between the two mounting posts and the distal trachea end was also secured. The mounted trachea was then gently stretched using the chamber mounting-posts spacing control to the previously measured *in vivo* intercartliage width for rats of this size. The chamber lid was secured onto the tissue bath and a small volume (~50 ml) of PBS was added to the chamber to maintain humidity and prevent the trachea from drying out during imaging. The fluid level remained below the trachea to ensure that fluid could not enter the tracheal lumen if the seal at the trachea mounting points connecting was poor. PBS was used to ensure that during chamber mounting into the beam that any splashes of the solution onto the trachea would be of the relevant ionically-compatible media used in trachea preparation. The humidity of the air surrounding the trachea inside the chamber was measured using a sensitive hygrometer (Lutron HT-3017).

### Marker particle delivery

MCT marker particles with a diameter of 20 μm (Corpuscular, NY, USA) were composed of alumina (Al_2_O_3_), and were either uncoated (n = 6), or their surface was carboxylated (COOH) (n = 10) or aminated (NH_2_) (n = 5). After the trachea was positioned, a sample of one type of particle was delivered to the lumen of the trachea as previously described^11^ using a fine cannula attached to a pressured air source via an electronically actuated valve. Briefly, the tip of the cannula was pushed into the bottom of a ~2 mm deep particle sample held in a small microfuge tube and the outside of the cannula was carefully wiped clean, leaving a loosely-packed filled internal pellet of particles ready for delivery. The cannula was then fed through the connecting tube (shown in Fig. 7), until the tip of the cannula was just inside the trachea. The particles were delivered using a computer interface that opened the valve controlling air pressure for a period of 10 ms, dispersing the particles onto the length of the tracheal surface.

### Pre-imaging setup and MCT imaging

The inspiratory and expiratory tubes of a small animal ventilator (AccuVent 200, Notting Hill Devices, Australia) were joined together with a Y-adaptor, and this was connected to the tissue bath connecting tube that was attached to the proximal end of the trachea. To simulate ventilation, the ventilator was configured to provide a PIP of 3 cmH_2_O, and a PEEP of −2 cmH_2_O, with inspiratory and expiratory durations of 500 ms. The ventilator air supply was bubbled to humidify it to 100% using a stainless steel sparging block with 2 μm holes (Keg King, Australia) to produce extremely fine bubbles. The humidity of the ventilated gas was measured with the hygrometer (Lutron HT-3017). An Aeroneb aerosol generator (Aerogen, Galway, Ireland) was also located in the inspiratory line. The connecting tube on the distal end of the trachea was left open to atmosphere. This configuration meant that the trachea was never pressurised by the ventilator, but that the air supply oscillated to simulate respiration, with a small flow bias to facilitate aerosol delivery to the trachea.

The ventilator output timing signals were used to trigger capture of 3 images per breath; one at the start of inspiration, one during PIP (the breath hold phase), and one during PEEP (the expiratory phase). The length of the entire imaging period was 20 minutes. After 2 minutes of baseline imaging aerosolised HS was applied to the tracheal lumen by activating the Aeroneb (60 actuations with inspiratory at 75 ms/breath). The desired HS treatment delivery was simultaneously monitored using an optical sensor for aerosol presence, and a webcam (Fig. 7b).

### Post experiment analysis

All MCT bead tracking and analysis was conducted using previously developed custom software written in Matlab (MathWorks,USA), in which a user manually tracks moving beads^9^. Briefly, all images were flat and dark field corrected. The user is then shown a randomly selected sequence of 15 seconds worth of frames, from which individual particles can be tracked between consecutive frames. To make the process more efficient, only particles that appeared to move in this sequence of images were tracked; stationary particles were neither identified nor tracked and were hence excluded from measurements. The user determined whether a particle was moving or stationary, based on the particle displacement for the frame sequence duration.

From the detected particle motion the velocity of the particles (and hence the MCT rate) was measured. Similar to previous work, for each time point and for each animal the mean velocity magnitude of every particle was calculated^9,16^. As was used by Hoegger *et al*.^6^, the maximum number of particles that moved for each trachea was calculated to evaluate whether the bead coating affected the ability of the mucus to clear the particles. For each trachea the frame containing the maximum number of particles moving was determined from the manual tracking results; the proportion of particles that were moving (as a percentage of the total number of particles in a given frame) were then calculated. This proportion was calculated using the number of moving particles from the manual tracking results and by manually counting the total number of particles in that frame.

## Supplementary information

Supplementary Video S1

## Data Availability

The datasets analysed during the current study are available from the corresponding author on reasonable request.
